# Non – biopsy methods to determine hepatic fibrosis

**Published:** 2009-11-25

**Authors:** C Fierbinteanu–Braticevici, M Purcarea

**Affiliations:** Medical Clinic Ⅱ and Gastroenterology, University Hospital Bucharest Romania

**Keywords:** fibrosis, liver biopsy, serum markers, transient elastography, recurrency treatment

## Abstract

The prognosis and clinical management of chronic liver diseases are highly dependent on the extent of liver fibrosis. Bigger the fibrosis, worse the prognosis; and bigger the risk of progression to cirrhosis.  In current practice, liver biopsy is most frequently performed to assess the grade of inflammation and stage of fibrosis thereby providing prognostic information on which to base treatment decisions upon.

Liver biopsy is becoming more and more useless in the management of chronic liver disease due to large sampling error, consistent inter–observer disagreement, high emotional cost of patient, enormous health care commitment in case of rare but possible severe complications, the fact that it is a snapshot of a process that is everything but a frozen one. Therefore, every methodology that avoids performing this invasive procedure is welcome.

The purpose of this article is to present the noninvasive evaluation of patients with chronic liver disease as an alternative of liver biopsy in the assessment of hepatic structure and function.

Hepatic fibrosis and cirrhosis are the endpoints of most types of chronic liver disease and the result of replacement of liver tissue with collagenous scar. The liver responds to injury with wound healing and, subsequently, fibrosis. This response occurs after essentially all kinds of injury (e.g. from virus, alcohol, iron, copper). Fibrosis is a result of an imbalance between fibrolytic and fibrogenic processes.  Hepatic stellate cells (HCSs) are the key effector of cell fibrogenesis. In the normal liver HCSs are quiescent, but with liver injury they are activated and transform into myofibroblast–like cells, capable of proliferation. In addition, HSCs are an important source of metalloproteinase (TIMPs), matrix–degrading proteases with a central role in the remodeling of extracellular matrix.

Progressive scarring in response to a persisting liver insult leads to ‘cirrhosis’ characterized by fibrotic bands, parenchymal nodules and vascular distortion. Hepatic fibrosis and cirrhosis are morphologically defined and the pattern and extent of the morphological changes depend on the cause and stage of fibrosis. Accordingly, there is a wide spectrum in the degree of fibrosis and in the severity of clinical symptoms. Clinical presentation may vary widely, ranging from absent or nonspecific symptoms to life threatening ones. In most cases, no clear dividing line can be drawn between cirrhosis and the preceding liver disease because the transition is gradual and unapparent.

## Indications for assessing liver fibrosis

It is important to have a safe and effective diagnostic tool for liver fibrosis for several reasons. Firstly, fibrosis is a central parameter of the severity of chronic liver disease associated with liver morbidity and mortality. Secondly, fibrosis is a key predictor for further progression to cirrhosis. Thirdly, advanced stage of fibrosis is the major criterion to start causal treatment. 

For years, liver fibrosis was considered irreversible; however, there is accumulated clinical and experimental evidence to suggest that this axiom should be rejected. Reversal of fibrosis is a reality in some cases. Existing treatments, particularly those that treat the primary injury, can allow the complete resolution. When the underlying insult can be removed, it may soon be possible to offer patients specific antifibrotic therapy to reverse liver damage. 

Assessing liver fibrosis is relevant for validation and monitoring any antifibrotic therapy. If compared to other prognostic parameters, fibrosis is definitely more important than liver inflammation and liver steatosis

## Options for Liver fibrosis assessment

### Percutaneous Liver biopsy

Limitations of liver biopsy

**Fig 1:**
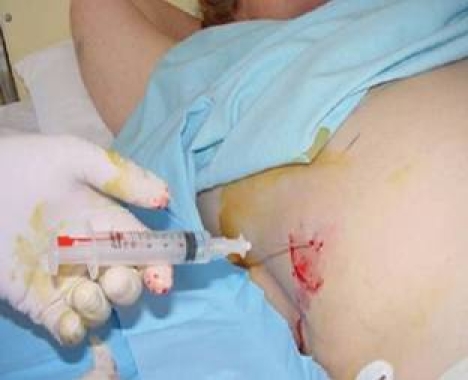
Percutaneous Liver Biopsy (Menghini)

Although liver biopsy is often called the gold standard for assessment of liver disease, the true standard is the clinical outcome or what happens to the patient. Liver biopsy was initially developed as a diagnostic tool to help determine the cause of liver dysfunction. In some instances, liver biopsy is performed to determine the effect of treatment of known liver disease. It is an invasive procedure with certain unavoidable risks and complications. Significant complications occur in 1–5% of patients and the mortality rate is reported to be 1:1000 and 1/10.000 (Table 1). Despite these reservations, needle liver biopsy remains the primary tool in diagnosing liver diseases and in staging liver fibrosis (Fig 1).

**Table 1:** Contraindications to percutaneous Liver Biopsy

Uncooperative patient
Bleeding disorder
Infection of skin, pleura, right lower lung or peritoneum overlying the liver
Suspected liver abscess or vascular lesion
Difficulty in determining liver location, as with ascites
Severe extrahepatic obstruction

Needle liver biopsy, however, removes only about 1/50.000 of the liver and so carries substantial sampling error. Both autopsy and laparoscopic studies have clearly shown that cirrhosis is missed on a single blind liver biopsy in 10–30% of cases. Both the size of the biopsy and the number of biopsies taken have a major effect on accuracy. An adequate biopsy should be at least 15 mm in length and contain more than 5 portal tracts. Studies have shown that biopsy specimens less than 25 mm in length can lead to underdiagnosis of cirrhosis; therefore, some investigators recommend larger biopsies. The problem of sampling error is compounded because liver biopsies are more and more often performed using the transjugular or radiographically guided approach, by which smaller samples are obtained.

Several studies have investigated the inter–observer and intra–observer variability in the histological and pathologic diagnosis of liver fibrosis based on biopsy specimens. Staging scores for fibrosis such as METAVIR, Ishak and Scheuer systems were created to standardize the evaluation of liver biopsies to minimize observer variation. Although not as great as the errors attributed to sampling variability, errors in disease staging for fibrosis with a 1 METAVIR stage appear occur in up to 20% of patients and a misdiagnosis of cirrhosis in 15% of patients. Staging errors especially for therapeutic decisions can lead to under treatment. This is particularly true for METAVIR stage 2 patients with chronic hepatitis C. Considering non–invasive tests, it is important to realize that the comparator liver biopsy is wrong in 20 % of cases, particularly where there is intermediate stage disease.  More problems with histology are:

- fibrosis progression in the majority of patients is slow, from normal to cirrhosis (stage 0 to stage 4 in >20 years).
- follow–up biopsy is too insensitive to detect changes in fibrosis progression or regression within weeks to months or even years.

To quantify fibrosis more accurately, automated morphometry was investigated. Although the quantity of fibrosis detected with morphometry correlates to the stages of fibrosis, the nature of this relationship is not linear. As technology continues to develop, incorporation of topography and quantification of fibrosis may increase the value of this automated technology.

### Noninvasive Tests

Noninvasive tests are an attractive alternative to hepatic biopsy in standardizing and monitoring chronic liver affections. This is the reason why the efforts to assess the lesions stage through non–invasive methods are justified. A noninvasive method which can provide the same information is also desired in the cases in which this technique cannot be possible. In a conventional way, lesion degree is assessed through tests that reflect the hepatic cells' permeability (transaminases) and the activity of hepatic cells synthesis (albumin, bilirubin, protrombin time). Noninvasive tests can be classified in several ways based on the modality of the test (serum blood tests or imaging) or the constituents of the tests (direct markers versus indirect markers of fibrosis). With the evolution of noninvasive tests, the performance can improve particularly with the use of combination or serial noninvasive tests. 

#### Serological assays

A large number of serological markers of liver hepatic fibrosis have been studied for their accuracy in staging hepatic fibrosis. The ideal fibrosis test would have a high sensitivity and specificity, be relatively inexpensive, reflect fibrosis irrespective of cause and be easy to perform being reproducible and easily interpreted. 

#### Direct serum markers of liver fibrosis

**Fig 2:**
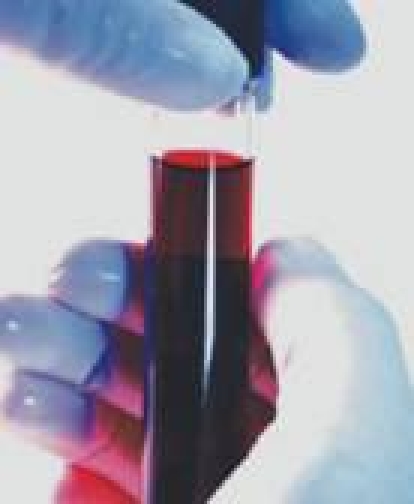
Serum markers of fibrosis

**Abbreviations**: TIMP–1, tissue inhibitor of metalloproteinase, GGT, gamma–glutamine transferase, ELFGA, European Liver Fibrosis Group algorithm, AST, aspartate aminotransferase, ALT, alanine aminotransferase (Table 2)

It has been suggested that measurement of direct serum markers of fibrogenesis, such as procollagen type Ⅲ N– terminal peptide (PIIINP) and direct serum marker of fibrolysis (MMP–1) might be helpful in evaluating liver fibrosis. Because of the lack of specificity, no marker of liver fibrosis has demonstrated test characteristics equivalent to liver biopsy(Fig 2).

**Table 2:** Serum indices of hepatic fibrosis

**FPI**	Age, cholesterol, insulin resistance, past alcohol use,AST
**Pohl Score**	Platelet, AST, ALT
**Fibrotest**	alpha_2_ macroglobulin, GGT, age, sex, haptoglobin, total bilirubin, apolipolipoptrtein A_1_
**Forns Index**	Age, platelet, GGT, cholesterol
**APRI Index**	AST, platelet
**Hepa Score**	Age, sex, hyaluronic acid, alpha_2_ macroglobulin, GGT
**ELF**	Propeptide Ⅱ collagen, haptoglobin, TIMP–1
**ELFGA**	Age,amino–terminal propeptide of type Ⅲ collagen, haptoglobin, TIMP–1
**FibroSpect Ⅱ**	Haptoglobin, TIMP–1, alpha_2_ macroglobulin

The results of all these tests are similar to ROC that show an area under the curve (AUC) of approximately 0,80–0,85. The clinical utility of these tests is to rapidly screen patients for the presence of mild or significant liver disease. They can prevent the need for liver biopsy in 40% of patients and can be followed up over time.

#### Indirect markers of liver fibrosis

A variety of indirect markers of liver fibrosis have been evaluated. Several simple ratios and indices have been developed using aminotransferases, platelet count, prothrombine time and age, such as the aspartate aminotransferase (AST)/alanine aminotransferase (ALT) ratio, the age–platelet index (AP index), the Pohl score, the cirrhosis determinant score (CDS) and the AST to platelet ratio index (APRI). Multivariate analyses have been directed at identifying markers of fibrosis among extracellular matrix molecules for combination into multicomponent serum panel or models with more testing capabilities. The most widely known of these are listed in biomarkers:

- rather represent the whole liver;
- only permit crude staging;
- rather reflect liver function (secretion, endothelial uptake). The results the serological tests are shown in Table 3 (adapted from Lai and Afdhal).

**Table 3:** Serological Tests for Liver Fibrosis; Abbreviations: PPV, positive predictive value, NPV, negative predictive value

	Patients	Serum Markers	AUROC (95%CI)	Sens.	Spec.	PPV	NPV
Wai et all	192	**APRI**	0,88	41%	95%	88%	64%
Rosenberg et all	1021	**ELF**	0,80	90,5%	41%	99%	92%
Imbert–Bismut	339	**Fibrotest**	0,87	87%	59%	63%	85%
Castera et all	183	**Fibrotest**	0,88	NA	NA	NA	NA
Patel et all	402	**Fibrospect**	0,831	77%	73%	74%	76%
Adams et all	221	**Hepascore**	0,82	63%	89%	88%	95%

Future studies will focus on using these tests for prediction of clinical outcomes and for the risk of disease progression.

## Elastography

**Fig 3:**
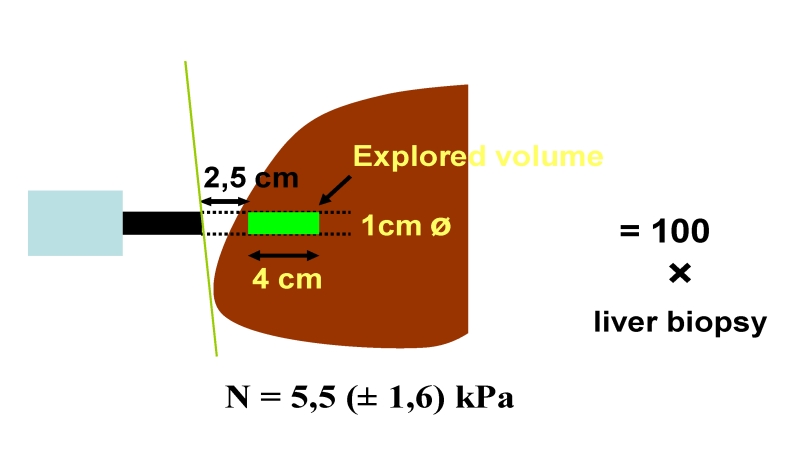
Fibroscan ‘The stiffer the liver, the faster the shear wave propagates’

Transient elastography is more sensitive than currently available radiologic techniques for staging hepatic fibrosis. This technique uses a probe, (Fibroscan, Echosens), which includes an ultrasonic transducer, that creates a vibration of low frequency (50MHz) and amplitude, which is transmitted into the liver. The vibration wave induces an elastic shear wave that propagates through the liver. The velocity of the wave, as it passes through the liver, correlates directly with tissue stiffness or elasticity; the propagated wave travels faster with increasing fibrosis. A pulse–echo ultrasound allows measurement of the wave velocity and the results are presented as kilopascals (kPa). Stiffness is measured within a cylinder, measuring 1 cm in width and 4 cm in length, producing an estimated sampling area that is 100 times greater than biopsy. (Fig 3).

The elasticity result is given as the median of 10 accurate measurements; results range from 10 to 90 kPa in various stages of chronic liver disease. This technology demonstrates many features desirable for the non– invasive assessment of hepatic fibrosis. It is painless, quick (5 min), safe, can be performed bedside. A major advantage of transient elastography is the ability to take multiple measurements in the same liver. It provides immediate results and only short training is necessary.

The theoretical limitation of transient elastography are primarily mechanical factors that produce poor propagation of the wave, including the thickness and type of tissue separating the liver from the transducer (marked obesity and ascites), the ‘window’ quality (the rib space may be too narrow to allow good wave propagation) and some hepatic tissue characteristics, such as fatty liver or liver inflammation.

*Ziol and collegues* enrolled 327 patients with chronic hepatitis C in a multicenter study comparing METAVIR liver fibrosis stages on biopsy specimens with transient elastography. It is the largest study of hepatic elastography reported which concluded that elastography is a reliable tool to detect significant fibrosis or cirrhosis.

*Castera and collegues* studied 183 consecutive patients who had hepatitis C and compared the results of Fibroscan with FibroTest and the aspartate transaminase to platelet ratio (APRI) in their ability to detect cirrhosis. The investigators concluded that the tests had similar value in detecting cirrhosis, although the Fibroscan had the single best performance. The authors conclude that liver biopsy could be avoided in most patients with hepatitis C.

Another study, by *Foucher et all.*, was carried out on 711 patients with chronic liver disease from all etiologies. Of the 711 patients, 354 patients had a liver biopsy. Foucher et all, found transient elastography to be of value in predicting fibrosis; it also correlates with complications of cirrhosis such as esophageal varices and bleeding, ascites and hepatocellular carcinoma. The results of many of these studies are shown in Table 4.

**Table 4:** Performance of transient elastography; Abbreviations: HCV, hepatitis C virus, NAFLD, nonalcoholic fatty liver disease, signf., significant

Study	Disease	Prevalence of sign of fibrosis	AUC	Threshold kPa	Sensitivity	Specificity
Fraquelli et al	Mixed	50	0,86	7,6	81%	76%
Gomez–Dominiguez	Mixed	82	0,74	4,0	94%	33%
Chang et al.	Mixed	44	0,86	9,0	83%	85%
Castera et al.	HCV	74	0,83	7,1	67%	89%
Ziol et al.	HCV	65	0,79	8,8	56%	91%
Yoneda et al.	NAFLD	49	0,87	6,6	83%	81%

An alternative technique is to use MR elastography which has the advantage of being able to examine more parts of the liver including both lobes although it is significantly more expensive and time consuming.

## Future Trends

Proteomics, genomics, genetic risk profiling and breath tests are exciting new technologies under investigation. Incorporation of non–invasive tests into large natural history cohort studies and into therapeutic trials should be a priority in the next years.

## Conclusions

- Advanced fibrosis is the major predictor of morbidity and mortality of chronic liver disease.
- Sampling variability limits the usefulness of liver biopsy to stage fibrosis.
- Current biomarkers scores can spare up to 40% of patients with F0–F1 liver biopsy.
- Clinical proof and monitoring the antifibrotic drug effects require better noninvasive tests for fibrosis and especially for the dynamics of fibrogenesis
- Transient elastography is a very promising noninvasive method for the diagnosis of significant fibrosis in patients with chronic liver disease.
- Combining transient elastography with serum markers (FibroTest) as first line assessment could avoid liver biopsy in the majority of these patients.
- Transient elastography is currently the most accurate method for the diagnosis of cirrhosis.
- Because of its excellent acceptance by patients, transient elastography could be useful for monitoring fibrosis.
- Guidelines are needed for its use in clinical practice.

## References

[1] Darby IA, Hewitson TD (2007). Fibroblast differentiation in wound healing and 
fibrosis. Int Rev Cytol.

[2] Friedman SL (2008). Hepatic stellate cells: protean, multifunctional, 
and enigmatic cells of the liver. Physiol Rev.

[3] Laleman W, Van Landeghem L, Severi T (2007). Both Ca21–dependent and –independent 
pathways are involved in rat hepatic stellate cell contraction 
and intrahepatic hyper responsiveness to methoxamine. Am J Physiol Gastrointest Live Physiol.

[4] Vanheule E, Geerts AM, Reynaert H (2008). Influence of somatostatin and octreotide on 
liver microcirculation in an experimental mouse model of cirrhosis 
studied by intravital fluorescence microscopy. Liver Int.

[5] Melton AC, Datta A, Yee HF (2006). Ca21]i–independent contractile force generation 
by rat hepatic stellate cells in response to endothelin–1. Am J Physiol Gastrointest Liver Physiol.

[6] Nakamura K, Koga Y, Sakai H (2007). cGMP–dependent relaxation of smooth muscle 
is coupled with the change in the phosphorylation of myosin phosphatase. Circ Res.

[7] Laleman W, Van Landeghem L, Van der Elst I (2007). Nitroflurbiprofen, a nitric oxidereleasing 
cyclooxygenase inhibitor, improves cirrhotic portal hypertension in rats. Gastroenterology.

[8] Thein HH, Yi Q, Dore GJ (2008). Estimation of stage–specific fibrosis 
progression rates in chronic hepatitis C virus infection: 
a meta–analysis and meta–regression. Hepatology.

[9] Ryder SD, Irving WL, Jones DA (2004). Progression of hepatic fibrosis in patients with 
hepatitis C: a prospective repeat liver biopsy study. GUT.

[10] Cadranel JF, Rufat P, Degos F (2000). Practices of liver biopsy in France: results of 
a prospective nationwide survey. For the Group of Epidemiology of 
the French Association for the study of the liver (AFEF). Hepatology.

[11] Regev A, Berho M, Jeffers LJ (2002). Sampling error and intra–observer variation in 
liver biopsy in patients with chronic HCV infection. Am J Gastroenterol.

[12] The French METAVIR Cooperative Study Group (1994). 12.	Intra–observer and inter–
observer variations in liver biopsy interpretation in patients 
with chronic hepatitis C. Hepatology.

[13] Bedossa P, Dargere D, Paradis V (2003). Mutations in the p53 gene occur in diverse human 
tumour types. Hepatology.

[14] Wright M, Thursz M, Pullen R (2003). Quantitative versus morphological assessment of 
liver fibrosis: semi–quantitative scores are more robust 
than digital image fibrosis area estimation. Liver Int.

[15] Standish RA, Cholongitas E, Dhillon A (2006). An appraisal of the histopathological assessment of 
liver fibrosis. Gut.

[16] Deeks J (2006). Evaluations of diagnostic and screening tests. BMJ.

[17] Guha IN, Parkes J, Roderick P (2008). Noninvasive markers of fibrosis in nonalcoholic fatty 
liver disease: validating the European Liver Fibrosis Panel and 
exploring simple markers. Hepatology.

[18] Parkes J, Guha IN, Roderick P (2006). OPerformance of serum marker panels for liver fibrosis 
in chronic hepatitis C. J Hepatol.

[19] Gebo KA, Herlong HF, Torbenson MS (2002). Role of liver biopsy in management of chronic hepatitis 
C: a systematic review. Hepatology.

[20] Kaul V, Friedenberg FK, Braitman LE (2002). Development and validation of a model to diagnose 
cirrhosis in patients with hepatitis C. Am J Gastroenterol.

[21] Huang H, Shiffman ML, Cheung RC (2006). Identification of two gene variants associated with risk 
of advanced fibrosis in patients with chronic hepatitis C. Gastroenterology.

[22] Morra R, Munteanu M, Bedossa P (2007). CDiagnostic value of serum protein profiling 
by SELDI–TOF ProteinChip compared with a biochemical 
marker, FibroTest, for the diagnosis of advanced fibrosis in patients 
with chronic hepatitis C. Aliment Pharmacol Ther.

[23] Poon TC, Hui AY, Chan HL (2005). Prediction of liver fibrosis and cirrhosis in 
chronic hepatitis B infection by serum proteomic fingerprinting: a 
pilot study. Clin Chem.

[24] Callewaert N, Van Vlierberghe H, Van Hecke A (2004). Noninvasive diagnosis of liver cirrhosis using 
DNA sequence–based total serum protein glycomics. Nat
Med.

[25] Lim AK, Taylor–Robinson SD, Patel N (2005). Hepatic vein transit times using a micro bubble agent 
can predict disease severity non–invasively in patients 
with hepatitis C. Gut.

[26] Bolkenius U, Hahn D, Gressner AM (2004). OGlucocorticoids decrease the bioavailability 
of TGF–beta which leads to a reduced TGF–beta signaling 
in hepatic stellate cells. Biochem Biophys Res Commun.

[27] Coco B, Oliveri F, Maina AM (2007). Transient elastography: a new surrogate marker of 
liver fibrosis influenced by major changes of FibroScan in a 
prospective study of transaminases. J Viral Hepat.

[28] Cobbold JF, Taylor–Robinson sd (2008). Transient elastography in acute hepatitis: 
all that's stiff is not fibrosis. Hepatology.

[29] Foucher J, Castera L, Bernard PH (2006). Prevalence and factors associated with failure of 
liver stiffness measurement using FibroScan in a prospective study of 
2114 examinations 5. Eur J Gastroenterol Hepatol.

[30] 	Romero–Gomez M, Gomez–Gonzalez E, Madrazo A (2008). Optical analysis of computed tomography images of the 
liver predicts fibrosis stage and distribution in chronic hepatitis C. Hepatology.

[31] Boulanger Y, Amara M, Lepanto L (2003). Diffusion–weighted MR imaging of the liver 
of hepatitis C patients. NMR Biomed.

[32] Girometti R, Furlan A, Bazzocchi M (2007). Diffusion–weighted MRI in evaluating liver 
fibrosis: a feasibility study in cirrhotic patients. Radiol Med.

[33] Aguirre DA, Behling CA, Alpert E (2006). Liver fibrosis: noninvasive diagnosis with double 
contrast material–enhanced MR imaging. Radiology.

[34] Lim AK, Patel N, Hamilton G (2003). The relationship of in vivo 31P MR spectroscopy 
to histology in chronic hepatitis C. Hepatology.

[35] Huwart L, Sempoux C, Salameh N (2007). Liver fibrosis: noninvasive assessment with MR 
elastography versus aspartate aminotransferase–
to–platelet ratio index. Radiology.

[36] Shaheen AA, Myers RP (2007). Diagnostic accuracy of the 
aspartate aminotransferaseto– platelet ratio index for 
the prediction of hepatitis C–related fibrosis: a systematic 
review. Hepatology.

[37] Shaheen AA, Myers RP (2008). Systematic review and meta–analysis of 
the diagnostic accuracy of fibrosis marker panels in patients 
with HIV/hepatitis C coinfection. HIV Clin Trials.

[38] Shaheen AA, Wan AF, Myers RP (2007). FibroTest and FibroScan for the prediction of 
hepatitis C–related fibrosis: a systematic review of 
diagnostic test accuracy. Am J Gastroenterol.

[39] Poynard T, Imbert–Bismut F, Munteanu M (2004). Overview of the diagnostic value of biochemical markers 
of liver fibrosis (FibroTest, HCV FibroSure) and necrosis (ActiTest) 
in patients with chronic hepatitis C. Comp Hepatol.

[40] Guha IN, Parkes J, Roderick PR (2006). Non–invasive markers associated with liver 
fibrosis in non–alcoholic fatty liver disease. Gut.

[41] Talwalkar JA, Kurtz DM, Schoenleber SJ (2007). Ultrasound–based transient elastography for 
the detection of hepatic fibrosis: systematic review 
and meta–analysis. Clin Gastroenterol Hepatol.

[42] Sebastiani G, Vario A, Guido M (2006). Stepwise combination algorithms of noninvasive markers 
to diagnose significant fibrosis in chronic hepatitis C. J Hepatol.

[43] Castera L, Vergniol J, Foucher J (2005). Prospective comparison of transient 
elastography, FibroTest, APRI and liver biopsy for assessment of 
fibrosis in chronic hepatitis C. Gastroenterology.

[44] Ziol M, Handra–Luca A, Kettaneh A (2005). Noninvasive assessment of liver fibrosis by measurement 
of stiffness in patients with chronic hepatitis C. Hepatology.

